# Backbone NMR assignments of the extensive human and chicken TRPV4 N-terminal intrinsically disordered regions as important players in ion channel regulation

**DOI:** 10.1007/s12104-022-10080-9

**Published:** 2022-04-22

**Authors:** Benedikt Goretzki, Frederike Tebbe, Sarah-Ana Mitrovic, Ute A. Hellmich

**Affiliations:** 1 Faculty of Chemistry and Earth Sciences, Institute of Organic Chemistry and Macromolecular Chemistry and Cluster of Excellence “Balance of the Microverse”, Friedrich Schiller University Jena, Humboldtstrasse 10, 07443 Jena, Germany; 2 Center for Biomolecular Magnetic Resonance, Goethe-University, Max-von-Laue-Strasse 9, 60438 Frankfurt, Germany; 3 Department of Chemistry, Division Biochemistry, Johannes-Gutenberg-University Mainz, Johann-Joachim Becher-Weg 30, 55128 Mainz, Germany

**Keywords:** Transient receptor potential, TRP vanilloid, Ion channel, Intrinsically disordered protein, Regulatory domain, Structural dynamics

## Abstract

Transient receptor potential (TRP) channels are important pharmacological targets due to their ability to act as sensory transducers on the organismic and cellular level, as polymodal signal integrators and because of their role in numerous diseases. However, a detailed molecular understanding of the structural dynamics of TRP channels and their integration into larger cellular signalling networks remains challenging, in part due to the systematic absence of highly dynamic regions pivotal for channel regulation from available structures. In human TRP vanilloid 4 (TRPV4), a ubiquitously expressed homotetrameric cation channel involved in temperature, osmo- and mechano-sensation and in a multitude of (patho)physiological processes, the intrinsically disordered N-terminus encompasses 150 amino acids and thus represents > 17% of the entire channel sequence. Its deletion renders the channel significantly less excitable to agonists supporting a crucial role in TRPV4 activation and regulation. For a structural understanding and a comparison of its properties across species, we determined the NMR backbone assignments of the human and chicken TRPV4 N-terminal IDRs.

## Biological Context

Transient Receptor Potential (TRP) proteins are eukaryotic cation channels that play important roles in cellular homeostasis, pain and temperature sensation or host-pathogen interactions (González-Ramírez et al. 2017; Samanta et al. 2018; Spix et al. 2020). In mammals, there are six TRP subfamilies. The members of the TRP Vanilloid (TRPV) subfamily are further categorized into group I members (TRPV1-V4) and group II members (TRPV5 and V6). TRPV1-V4 are ‘ThermoTRPs’, channels that are activated by warmth and heat but generally act as diverse stimulus-activated non-selective cation channels, while TRPV5 and V6 are constitutively active, highly selective Ca^2+^ channels involved in systemic Ca^2+^ resorption (van Goor et al. 2020). TRP channels in general are capable of integrating diverse stimuli, but the ubiquitously expressed TRP Vanilloid 4 (TRPV4) protein displays exceptional polymodality even within the diverse TRP superfamily (White et al. 2016). TRPV4 is activated by moderate heat (Güler et al. 2002), osmotic and mechanical stress (Fernandes et al. 2008; Strotmann et al. 2000), pH (Suzuki et al. 2003), ions (Loukin et al. 2015), nucleotides (Phelps et al. 2010), lipids (Garcia-Elias et al. 2013; Takahashi et al. 2014), lipid metabolites (Watanabe et al. 2003) and lipid-like compounds (Watanabe et al. 2002), proteins (Cuajungco et al. 2006; D’hoedt et al. 2008; Doñate-Macián et al. 2018; McCray et al. 2021), plant-derived natural products (Ma et al. 2012; Peixoto-Neves et al. 2015; Smith et al. 2006), and small organic molecules (Garcia-Elias et al. 2014). TRPV4 is involved in numerous physiological functions ranging from neuritogenesis (Jang et al. 2012), bone and cartilage formation (Muramatsu et al. 2007), response to viral infections (Doñate-Macián et al. 2018), to the maintenance of the epidermal barrier (Sokabe et al. 2010), including in the lung (Weber et al. 2020) where its potential as a pharmacological target for the treatment of COVID-19 is currently being discussed (Kuebler et al. 2020).

While there has been significant progress regarding TRP channel structural characterization, including cryo-electron microscopy (cryo-EM) structures of human and frog TRPV4 (Botte et al. 2020; Deng et al. 2018), many questions regarding the structural basis of channel functional regulation remain. This is partly because the large intrinsically disordered regions in the channel N- and C-termini, which harbor many important interaction sites for regulatory proteins and lipids, are often not resolved or are intentionally removed from protein constructs used for structural studies (Goretzki et al. 2021; Hellmich and Gaudet 2014a). However, these ‘missing’ regions not only define TRP channel subfamily affiliation, but also present important lipid and protein interaction sites pivotal for channel regulation (Goretzki et al. 2021; Hellmich and Gaudet 2014b). The cytosolic N-terminal domain (NTD) of group I TRPV channels is composed of an α-helical ankyrin repeat domain preceded by an intrinsically disordered region (IDR). In TRPV4, the NTD acts as the central recruitment hub for regulatory partners such as ATP (Inada et al. 2012; Phelps et al. 2010), PIP_2_ (Garcia-Elias et al. 2013; Takahashi et al. 2014) or proteins such as PACSIN3 and RhoA (Cuajungco et al. 2006; D’hoedt et al. 2008; Goretzki et al. 2018; McCray et al. 2021). While the structural basis of TRPV4 (de)sensitization remains unknown, dysregulated interactions in the channel termini can affect channel response to incoming stimuli and ultimately determine cellular fate leading to e.g., neurite outgrowth or axonal degeneration (McCray et al. 2021; Woolums et al. 2020).

As opposed to structured proteins locking onto each other via complementary surfaces, intrinsically disordered proteins can engage in ‘one-to-many-signalling’ as a prerequisite for the participation in regulatory cascades and cellular protein networks. Therefore, intrinsic disorder is particularly prevalent in membrane receptors (Kjaergaard and Kragelund 2017). In TRP channels, IDRs typically present between a quarter and more than half of the entire protein sequence and endow the channels with the ability to dynamically react to environmental changes by undergoing transient protein-protein and protein-lipid interactions (Goretzki et al. 2021). With ~ 150 amino acids representing > 17% of the entire channel sequence, the N-terminal IDR of human TRPV4 is the largest in the TRPV subfamily. Its deletion renders the channel significantly less excitable to agonists supporting a crucial role in TRPV4 activation and regulation (Botte et al. 2020). For a better understanding of channel and species-specific channel regulation, we determined the backbone NMR assignments of the N-terminal IDRs of human and chicken TRPV4, which are 147 and 133 amino acids in length, respectively. The two proteins have 57% sequence identity (67% similarity) and the known regions important for channel regulation, i.e. a proline rich region and a PIP_2_ binding site (Cuajungco et al. 2006; D’hoedt et al. 2008; Garcia-Elias et al. 2013; Goretzki et al. 2018), are present in both.

## Methods and experiments

### Protein expression and purification

The DNA sequences encoding for the TRPV4-IDR from *H. sapiens* (human, hsTRPV4-IDR) and *G. gallus* (chicken, ggTRPV4-IDR) were cloned from cDNA into a pET11a vector with an N-terminal His_6_SUMO-tag via Gibson Assembly. Uniformly ^13^C, ^15^N-labeled TRPV4-IDR constructs were expressed in *E. coli* BL21-Gold(DE3) (Agilent Technologies) grown in M9 minimal medium supplemented with 0.1 mg/mL Ampicillin as well as ^15^N-labeled NH_4_Cl (0.75 g/L) and ^13^C-glucose (2 g/L) as the sole nitrogen and carbon sources. Protein expression was induced with 0.15 mM IPTG at an OD_600_ of 0.8 and cells were grown over night at 20 °C. After harvesting via centrifugation, cells were stored at -80 °C until further use. All purification steps were carried out at 4 °C. Harvested cells were resuspended in lysis buffer (20 mM Tris pH 8, 20 mM imidazole, 300 mM NaCl, 0.1% (v/v) Triton X-100, 1 mM DTT, lysozyme, DNAse, RNAse and protease inhibitor (Sigmafast)) followed by sonication on ice (Branson Sonifier 250). Cell debris was removed by centrifugation and the supernatant was applied to a Ni-NTA gravity flow column (Qiagen). After washing (20 mM Tris pH 8, 20 mM imidazole, 300 mM NaCl), the protein was eluted with 500 mM imidazole and dialyzed overnight (20 mM Tris pH 7, 300 mM NaCl, 1 mM DTT) in the presence of 5 mol % Ulp-1 protease. Afterwards, the cleaved proteins were separated from the His_6_SUMO-tag and residual uncleaved proteins via a reverse Ni-NTA affinity chromatography step and further purified via size exclusion chromatography (SEC) using a HiLoad prep grade 16/60 Superdex200 column (GE Healthcare) with 50 mM ammonium bicarbonate, 1 mM DTT as buffer. Purified, tag-free TRPV4-IDR constructs were lyophilized and stored at -20 °C until further use. For NMR measurements, the lyophilized powder was dissolved in the appropriate buffer.

## NMR spectroscopy

All NMR experiments were performed at 298 K on Bruker AVANCE III HD 600, 700, 800 and 900 MHz spectrometers equipped with cryogenic triple resonance probes (Bruker, Karlsruhe). Spectra for both the human and chicken TRPV4 construct were recorded at a concentration of 150–200 µM in 20 mM sodium phosphate pH 4.5, 150 mM NaCl, 1 mM DTT. The proton chemical shifts of ^13^C, ^15^N-labeled ggV4-IDR and hsV4-IDR were referenced to 2,2-dimethyl-2-silapentane-5-sulfonic acid (DSS) while the heteronuclear ^13^C and ^15^N chemical shifts were indirectly referenced with the appropriate conversion factors deposited in the BMRB. All spectra were processed with Bruker TopSpin™ 3.2 or 4.1. Backbone resonance assignments of ^13^C, ^15^N-ggTRPV4-IDR and ^13^C, ^15^N-hsTRPV4-IDR were carried out in CARA using HNCO, HN(CA)CO, HNCA and HNCACB triple resonance experiments. H^*α*^ resonances for secondary structure prediction were obtained using HBHACONH experiments. All experiments were recorded with standard Bruker pulse sequences including water suppression with WATERGATE.

## Disorder prediction

Sequence-based disorder predictions of hsTRPV4-IDR and ggTRPV4-IDR were obtained with the ODiNPred Server (https://st-protein.chem.au.dk/odinpred) (Dass et al. 2020). Secondary structure content was evaluated from secondary chemical shifts, calculated as the difference between random coil chemical shifts (RCCS) and the N, C’, C^*α*^, C^*β*^, H^*α*^, H^*β*^, H^*N*^ chemical shifts from the experimentally obtained backbone NMR resonance assignments of hsTRPV4-IDR and ggTRPV4-IDR. The theoretical RCCS values were determined with the POTENCI webserver (https://st-protein02.chem.au.dk/potenci/) (Nielsen and Mulder 2018). The per-residue secondary structure propensities (SSP) of the hsTRPV4- and ggTRPV4-IDR sequences were calculated from their C^*α*^, C^*β*^, H^*α*^ chemical shifts as described by Marsh et al. (Marsh et al. 2006) taking the NMR sample conditions (temperature, pH, and ionic strength) into account and excluding proline-preceding residues.

## Extent of assignment and data deposition

**Fig. 1:**
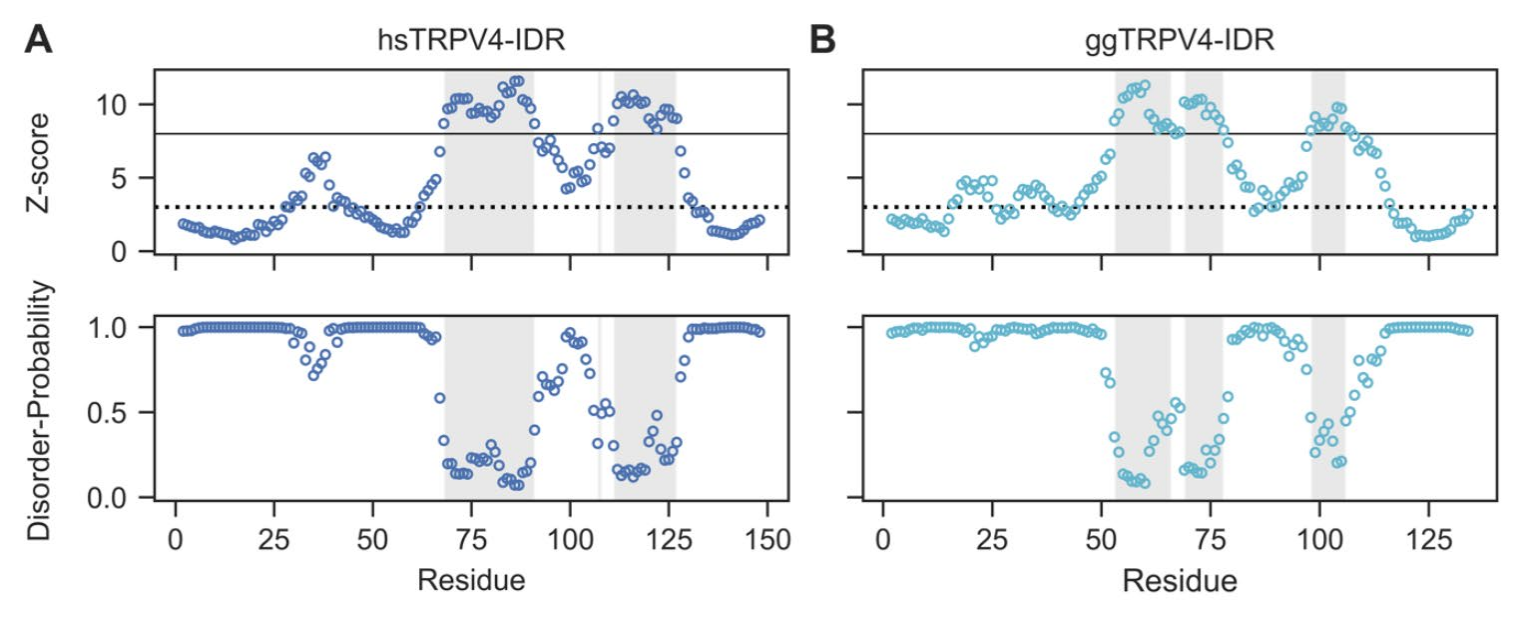
The sequence-based ODiNPred webserver predicts a significant amount of disorder in both the human (*Homo sapiens*, hs) and chicken (*Gallus gallus*, gg) TRPV4-IDR (A and B, respectively). However, several regions with low predicted disorder propensities indicate the formation of ordered structures within the TRPV4-IDR. The Z-score (upper panel) and disorder probability (lower panel) were calculated for each residue by ODiNPred (Dass et al. 2020). Residues with Z-score larger than 8 (solid line) are considered to be ordered while residues with Z-scores below 3 (dashed line) are fully disordered. Z-scores between 3 and 8 reflect transient structure formation. Regions with per-residue disorder propensities below 0.5 are shaded in light grey

While structural information on TRPV4 and its isolated N-terminal ankyrin repeat domain is available from X-ray and cryo-EM studies (Botte et al. 2020; Deng et al. 2018; Inada et al. 2012; Landouré et al. 2010; Takahashi et al. 2014), the intrinsically disordered region (IDR) preceding the ARD is consistently missing in high-resolution structures, because it was entirely or partially removed from the constructs used for structure determination. To estimate the extent of flexibility and disorder present in the TRPV4-IDR, we analyzed the human and chicken TRPV4-IDR sequences using ODiNPred (Dass et al. 2020), which assesses the per-residue disorder propensities based on a deep-neural network trained with NMR chemical shift data available through a greatly expanded version of the CheZOD database (Nielsen and Mulder 2016, 2020). The amino acid sequence based ODiNPred analysis predicts disorder probabilities larger than 0.5 for more than 70% of the residues in both the human and chicken TRPV4-IDR (Fig. 1 A, B). Nonetheless, 43 of 148 residues (29%) in the human and 33 of 133 residues (25%) in the chicken TRPV4-IDR have predicted disorder propensities of less than 0.5 (Fig. 1 A, B, shaded region). Notably, the conserved regions with low per-residue disorder probability in the TRPV4-IDR may exhibit (transient) structural order within the IDR, which could be functionally relevant in the context of the full-length TRPV4 channel.

**Fig. 2:**
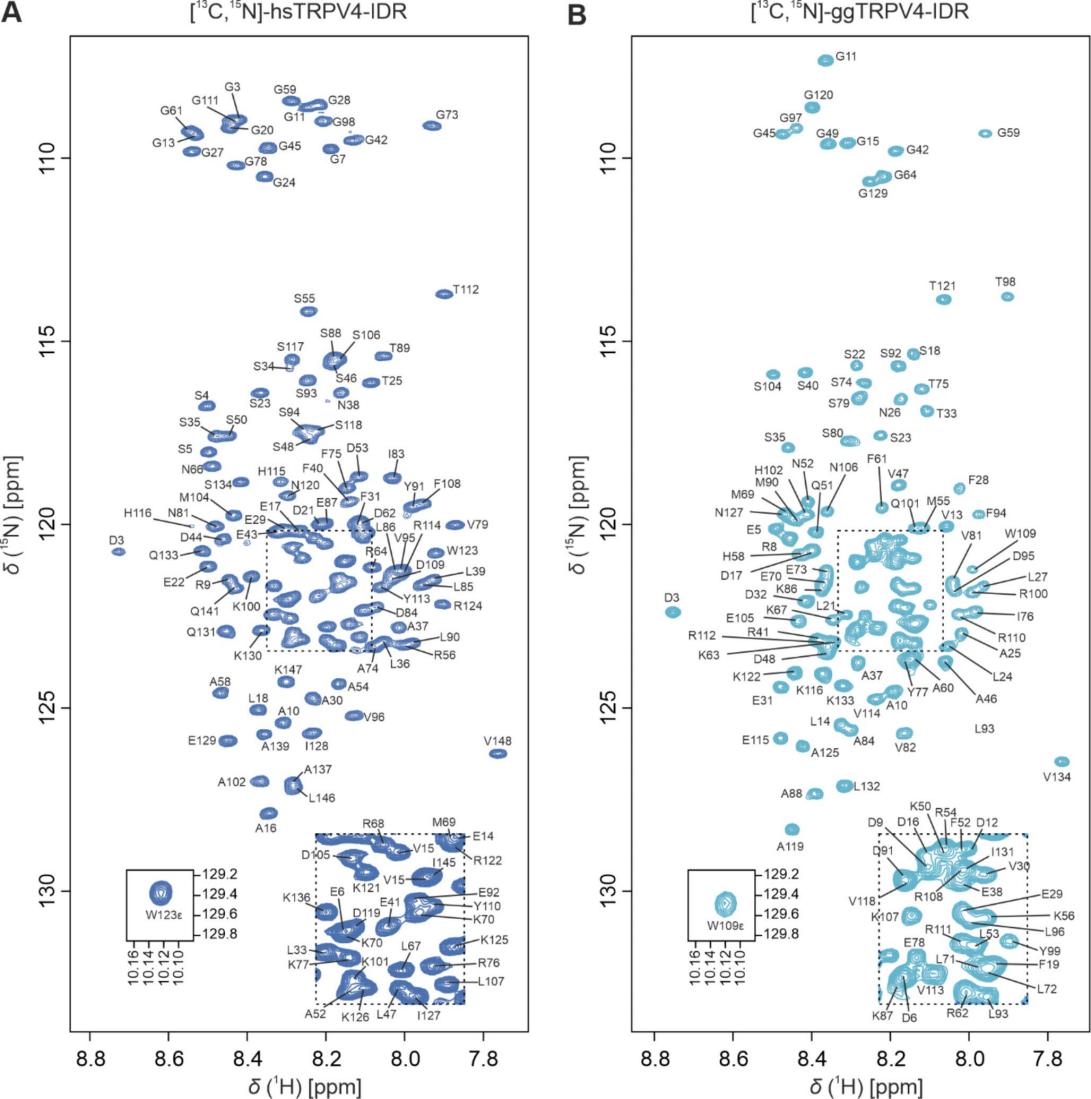
[^1^H, ^15^N]-TROSY-HSQC spectra of ^13^C, ^15^N-labeled human (A) and chicken (B) TRPV4-IDR (147 and 133 residues, respectively) in 20 mM NaP_i_, pH 4.5, 150 mM NaCl, 1 mM DTT, 0.1 mM DSS, 10% D_2_O at 298 K, recorded at 800 MHz. Assigned residues are annotated in one letter amino acid code according to the human and chicken full-length TRPV4 protein sequences (UniProtKB: Q9HBA0 and A0A1D5PXA5, respectively)

To analyze whether the sequence-based disorder prediction is accurate, we characterized the structure of the proximal TRPV4 N-terminus in solution experimentally, using full-length human and chicken TRPV4-IDR constructs (hsTRPV4-IDR and ggTRPV4-IDR). Only the first residue (M1) was missing in the final constructs used for backbone NMR assignments which thus comprise residues 2-134 of chicken and residues 2-148 of human TRPV4, respectively. In line with a low overall secondary structure content, the [^1^H, ^15^N]-TROSY-HSQC spectra of ^13^C, ^15^N-labeled human and chicken TRPV4-IDR (Fig. 2 A, B) show a narrow chemical shift dispersion. Presumably due to the absence of secondary structure, strong solvent exchange and subsequent line broadening at pH 7 and 298 K substantially hampered protein backbone NMR assignments of the IDR (data not shown). To suppress solvent exchange, all spectra were thus recorded at pH 4.5 with the standard set of triple-resonance NMR experiments (Fig. 2 A, B). Importantly, the decrease in pH did not affect the overall chemical shift dispersion in the [^1^H, ^15^N]-TROSY-HSQC spectra of both chicken and human TRPV4-IDR, thus indicating that the folding state of the proteins are not significantly influenced by the change in pH.

The human and chicken IDR contain 22 and 21 proline residues, respectively. For hsTRPV4-IDR, we could assign 95.7% of the backbone resonances (H^*N*^, N, C’, C^*α*^, C^*β*^). For ggTRPV4-IDR, the backbone resonance assignments are 94.5% complete. The C’, C^*α*^, C^*β*^ resonance assignments for proline residues are missing (*) in sequences with consecutive proline residues such as the triple proline motif P142*/P143*/P144 in hsTRPV4-IDR or P128*/P129*/P130 in ggTRPV4-IDR, respectively, or the double proline motifs P43*/P44 and P65*/P66 in ggTRPV4-IDR.

**Fig. 3:**
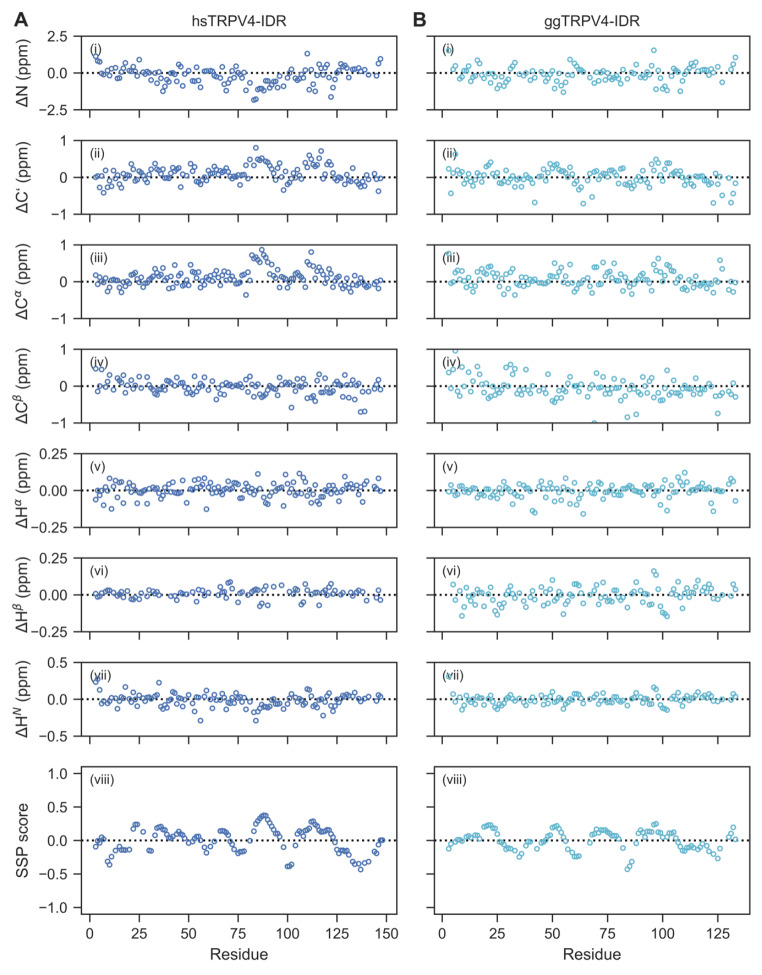
Chemical shift-based disorder analysis confirms that the human (A) and chicken (B) TRPV4-IDR are highly disordered throughout the entire protein sequence. (i-vii) Secondary chemical shifts calculated from the experimentally determined and predicted (using POTENCI, Nielsen and Mulder 2018) N, C’, C^*α*^, C^*β*^, H^α^, H^*β*^, H^*N*^ chemical shifts. (viii) Secondary structure prediction based on C^*α*^, C^*β*^, H^*α*^ chemical shifts using the SSP script (Marsh et al. 2006). Positive and negative values reflect α-helix and β-sheet propensities, respectively

The chemical shift assignments obtained for the human and chicken TRPV4-IDR were used for a structural analysis based on secondary chemical shifts. For this, the POTENCI tool (Nielsen and Mulder 2018) was used to predict random coil chemical shifts at our experimental conditions and the predicted shifts compared to those experimentally determined. Overall, the experimental H^*N*^, N, C’, C^*α*^, C^*β*^, H^*α*^ and H^*β*^ chemical shifts are in good agreement with the theoretical random coil chemical shift values determined for both proteins (Fig. 3 A, B, i-vii). The mean differences between the experimental and predicted chemical shift values for the human TRPV4-IDR are ΔN = -0.18 ± 0.60 ppm, ΔH^*N*^ = -0.01± 0.09 ppm, ΔC’ = 0.14 ± 0.47 ppm, ΔC^*α*^ = 0.11 ± 0.23 ppm, ΔC^*β*^ = -0.05 ± 0.31 ppm, ΔH^*α*^ = 0.00 ± 0.05 ppm, and ΔH^*β*^ = 0.01 ± 0.03 ppm. Likewise, the mean differences for chicken TRPV4-IDR are ΔN = 0.08 ± 0.54 ppm, ΔH^*N*^ = -0.00± 0.07 ppm, ΔC’ = 0.00 ± 0.24 ppm, ΔC^*α*^ = 0.07 ± 0.22 ppm, ΔC^*β*^ = -0.06 ± 0.29 ppm, ΔH^*α*^ = 0.00 ± 0.05 ppm, and ΔH^*β*^ = 0.00 ± 0.03 ppm.

The finding that both human and chicken TRPV4-IDR are highly disordered is further supported by the single residue-specific secondary structure propensities (SSP) calculated from the C^*α*^, C^*β*^ and H^*α*^ chemical shifts. Here, the different chemical shifts are accumulated into a single score that allows conclusions about (dis)order for each residue (Marsh et al. 2006) (Fig. 3 A, B viii). The SSP method predicts very low secondary structure propensities throughout the human and chicken TRPV4-IDR sequences (mean SSP values of 0.01 ± 0.18 and 0.01 ± 0.14, respectively). The overall secondary structure content (both α-helix and β-sheet) according to the SSP score is 15.6% for the humanTRPV4-IDR and 15.3% for the chicken TRPV4-IDR, thereby significantly lower than what was predicted using the solely sequence-based ODiNPred webserver (Fig. 1 A, B) (Dass et al. 2020). This highlights the importance of experimental methods to validate structure predictions. Notably, residual secondary structure propensities calculated by the SSP method for human and chicken TRPV4-IDR proteins may indicate the formation of local transient secondary structures e.g., at putative binding sites for TRPV4 regulators such as proteins and lipids.

Previous NMR-based structural analyses of the TRPV4-IDR were restricted to a disordered peptide comprising the PIP_2_ binding site (PBS) and the proline-rich region (PRR) of the chicken TRPV4-IDR (residues 105–134) (Goretzki et al. 2018). The current study expands this to the full-length TRPV4-IDRs of two organisms. The chemical shift perturbations and subsequent analyses indicate that the TRPV4 N-terminal region preceding the ARD is a highly disordered protein region with little structure propensity. Given that we find similar disorder content for both human and chicken TRPV4-IDR, it seems likely that intrinsic disorder in the TRPV4 N-termini is a conserved feature across species thus making these important regulatory channel regions inaccessible to X-ray crystallography or cryo-EM but interesting spectroscopic targets.

## Data Availability

The backbone assignments of the wildtype human and chicken TRPV4 N-terminal intrinsically disordered regions have been deposited in the BioMagResBank (www.bmrb.wisc.edu) under the accession numbers 51147 and 51172.
